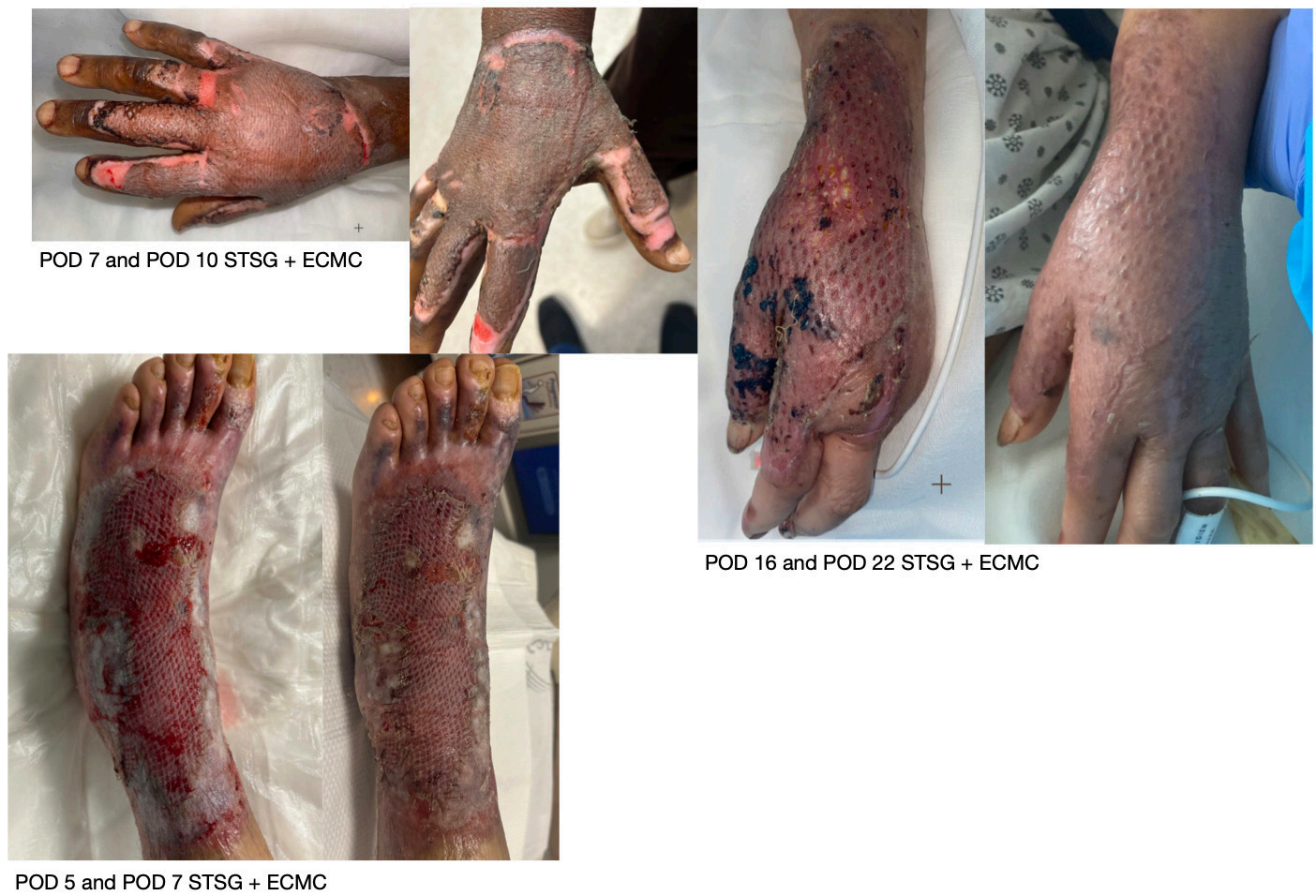# 539 Etherified Carboxymethyl Cellulose Matrix for Optimizing Hemostasis and Enhancing Graft Healing: A Case Series

**DOI:** 10.1093/jbcr/iraf019.168

**Published:** 2025-04-01

**Authors:** Maria Barahona, Hana Lopez-Quinones, Lily Cheng

**Affiliations:** Jacobi Burn Center; Jacobi Burn Center; Jacobi Burn Center

## Abstract

**Introduction:**

Surgical excision and autografting remain the standard of care for deep partial-thickness and full-thickness burns. Adequate hemostasis of the surgical wound bed is a key step for optimal graft healing. Etherified carboxymethyl cellulose (ECMC) matrix is a novel, all-natural, hemostatic dressing and wound healing matrix that provides rapid hemorrhage control. This work describes our experience with the use of ECMC on graft site wound beds to enhance hemostasis and optimize tissue regeneration.

**Methods:**

Data was collected from a retrospective electronic medical record review to obtain clinical photographs, demographic details, and postoperative outcomes. Patients had planned follow-ups of 2 months minimum. Descriptive statistics were used to summarize the findings.

**Results:**

A case series of five patients with deep partial to full-thickness burns was collected. The patient’s ages ranged from 36 years to 80 years with no previous history of burn injuries. Burn Total Body Surface Areas (TBSA) ranged from 5-20% resulting from contact, scald, or flame. All patients required surgical management that included sharp tangential excision and harvesting of split-thickness skin grafts with or without autologous skin cell suspension for definitive wound closure. ECMC was applied to the surgical wound beds for hemostasis before laying down the grafts. All wounds were covered with standard dressings that included a non-adherent contact layer with anti-microbial ointment, a moderately absorbent layer, and an outer compressive layer. Dressings were removed on postoperative day 5. All patients had adequate hemostasis achieved at the time of surgery and did not require post-operative interventions. The average time to re-epithelialization ranged from 7 to 10 days and was largely dependent on the depth of the burn with deeper injuries taking longer to heal. Dyschromia was minimal and there has been minimal evidence of hypertrophic scarring as seen in the average two-month follow-up.

**Conclusions:**

Using an etherified carboxymethylcellulose matrix helps promote rapid hemostasis, decreases blood loss, and enhances tissue regeneration. More extensive studies and long-term follow up are required but this work is a valuable stepping stone to identify key tools that can be used to improve patient cosmetic outcomes.

**Applicability of Research to Practice:**

Using an etherified carboxymethylcellulose matrix helps promote rapid hemostasis, decreases blood loss, and enhances tissue regeneration when performing split-thickness skin grafts with or without autologous skin cell suspension.

**Funding for the Study:**

N/A